# Post-traumatic stress disorder, psychiatric comorbidities and associated factors among refugees in Nakivale camp in southwestern Uganda

**DOI:** 10.1186/s12888-020-2480-1

**Published:** 2020-02-07

**Authors:** Achille Mwira Bapolisi, Suzan J. Song, Claire Kesande, Godfrey Zari Rukundo, Scholastic Ashaba

**Affiliations:** 1 Department of Psychiatry, Hôpital Provincial Général de Référence de Bukavu, Université Catholique de Bukavu, Bukavu, DR Congo; 2 Department of Psychiatry, Mbarara University of Science and Technology, Mbarara, Uganda; 3 Department of Psychiatry and Behavioural Sciences, George Washington University, Washington, DC USA

**Keywords:** Refugees, PTSD, Depression, Anxiety, Alcohol abuse, Pyschosocial needs, Uganda

## Abstract

**Background:**

Armed conflict in Africa has led to displacement of over 24.2 million people, more than 1.4 million of whom are living in Uganda. Studies show that refugees living in Ugandan refugee settlements are at increased risk for post-traumatic stress disorder. However data on the prevalence of other mental health problems among refugees including depression, anxiety and substance use disorder among refugees in Uganda is lacking. Our aim was to determine the prevalence of post-traumatic stress disorder, its main psychiatric comorbidities and perceived psychosocial needs among refugees in Nakivale refugee camp.

**Methods:**

We conducted a cross-sectional survey of refugee camp residents (*n* = 387) from nine different countries of origin. Psychiatric disorders were assessed using the MINI International Neuropsychiatric Interview (MINI) and perceived needs by the Humanitarian Emerging Settings Perceived Needs Scale (HESPER).

**Results:**

The prevalence of psychiatric disorders was high among refugees as was the level of perceived needs. The most prevalent psychiatric disorders were generalized anxiety disorders (73%), post-traumatic stress disorder (PTSD) (67%), major depressive disorder (58%) and substance use disorders (30%). There was a higher level of comorbidity between PTSD and substance use disorder (OR = 5.13), major depressive disorder (OR = 4.04) and generalized anxiety disorder (OR = 3.27).

In multivariate analysis, PTSD was positively associated with the perception of stress as a serious problem (OR = 6.52; *P*-value = 0.003), safety and protection for women in the community (OR = 2.35; P-value = 0.011), care for family (OR = 2.00; P-value = 0.035) and Place to live in (OR = 1.83; P-value = 0.04). After applying the Bonferroni correction, the perception of stress remained significantly associated with PTSD.

**Conclusion:**

Our findings suggest a strong association between PTSD, its main comorbidities and basic needs in Nakivale refugee camps. Mental health support should include psychological interventions as well as social assistance to improve the health of refugees.

## Background

There is an increasing number of displaced persons in the world with approximately 68.5 million people having been forced out of their homes; 25.4 million of these individuals are refugees [1]. Refugees are more likely to have experienced severe traumatic events in their home countries such as torture, rape, murder of family members, and have a higher incidence of post-traumatic stress disorder (PTSD) [2]. Most displaced persons and refugees are found in low and middle income settings, including sub-Saharan Africa which hosts more than 26% of the world’s refugee population [1].

Uganda is host to over 1.5 million refugees coming from Democratic Republic of Congo (44%), South Sudan (38%), Somalia (7%), Rwanda (8%) and Burundi (3%) [1]. Since 1998 the Democratic Republic of Congo has undergone successive war and armed conflicts that resulted in over 5.4 million deaths [3]. South Sudan has been devastated by civil war since December 2013 which resulted in about 400,000 deaths, 190,000 the direct result of fighting [4]. Likewise, a deadly civil war has ravaged Somalia since 1991 [5]. Burundi underwent a civil war from 1993 to 2005 which was followed by periods of political disturbances [6]. In Rwanda, in 1994, a genocide resulted in over 800,000 deaths in only three months [7]. Refugees tend to remain in Uganda for decades due to the extended nature of regional conflicts and their political aftermaths [8].

The refugees in Uganda are hosted in 11 main settlement camps (Achol-Pii Settlement, Bidi Bidi Settlement, Impevi Settlement, Kampala Settlement, Kiryandongo Settlement, Kyaka II Settlement, Kyangwali Settlement, Nakivale Settlement, Pagirinya Settlement, Rhino Settlement, Rwamwanaja Settlement). A considerable part of the refugees in Uganda (17%) are located in Nakivale camp in Insigiro District, south-western Uganda. Nakivale Refugee Settlement camp is characterized by a protracted situation in which refugee stay is not limited in time, assistance aims to enable local long term installation and self-reliance and refugees in Nakivale are allowed to move out the camp for business, work, recreation or any other reason [9]. Previous studies in Ugandan refugee camps have focused on PTSD and have shown a higher prevalence compared to other camps in the world. In northern Uganda, PTSD was found in 46% of Sudanese refugees and 18% of Ugandan nationals [10]. Among Somali community refugees, it was reported that 32% of adults suffer from PTSD [11]. Two previous studies in Nakivale refugee settlement, one among the Congolese reported PTSD prevalence of 61.7% [12]; and the second among the Rwandan and Somali refugees reported respectively 32 and 48.1% [13]. Daily life stressors in refugee camp settings may worsen PTSD and other psychiatric problems. Many studies have found a strong association between current stressors and mental health outcomes among traumatized populations [2, 14, 15]. This growing body of evidence has motivated the recommendation of psychosocial support and prioritizing addressing basic needs in addition to trauma-focused interventions [16]. Despite current efforts to address psychosocial needs of displaced persons, it is crucial to systematically analyse the vague concept of ‘current stressors’ in order to determine the main needs which are more likely to be associated with mental health outcomes among refugees. A clear understanding of driving factors can inform the efficient organization of scarce resources to help reduce suffering.

Prior studies done in Uganda evaluated specific nationalities, and were limited to PTSD and depression [12, 13, 17]. While prevalence of PTSD and Major Depressive Disorder (MDD) is shown to be high in refugee camp settings, knowledge of other psychiatric disorders and co-morbidities such as anxiety disorders and substance use disorder, can help guide treatment and intervention. Understanding the experience of various nationalities and, in turn, various cultures within the same context can be useful as well. Furthermore, understanding the association between basic psychosocial needs and psychiatric illness can help in the planning and provision of relevant psychosocial management and medical strategies for vulnerable populations.

## Methods

### Study design and setting

We conducted a cross sectional community survey in Nakivale Refugee Settlement in south-western Uganda. The camp hosts about 97,271 refugees of which 54% are female. There are nine nationalities of refugees in Nakivale camp including 44,270 (45.51%) Congolese, 26, 243 (26.91%) Burundians, 16,136 (16.59%) Somalis, 8933 (9.18%) Rwandans, 794 (0.81%) Ethiopians, 745 (0.77) Eritreans, 101 (0.10%) South-Sudanese, 21 (0.2%) Sudanese, 23 (0.02%) Kenyans and 5 (0.01%) Senegalese [1]. This setting was selected for its accessibility, large number of refugees and their representativeness regarding different nationalities.

### Participant recruitment

We used the Kish Leslie formula [18] to determine the sample size, considering a PTSD prevalence of 61.7% reported in a previous study among Congolese refugees in Nakivale camp [12]. The minimum sample size was 363 respondents. In order to account for incompleteness or loss of information, we increased the number by 7% to have a total of 387 respondents. Quota and stratified sampling were used to ensure representation of various nationalities in the camp. The strata consisted of 9 different nationalities present in the camp. The number of participants in each stratum was calculated proportionally from the representativeness of each nationality in the overall number of refugees in Nakivale. Hence, the sample constituted 176 Congolese, 104 Burundians, 64 Somalis, 36 Rwandans, 3 Ethiopian, 3 Eritreans, 1 Sudanese. In each stratum, the participants were randomly sampled from the registration list of the camp using random number generator in Excel. Eligible participants were adults refugees (recognized by the Ugandan Office of the Prime Minister under the 1951 Convention relating to the status of refugees), 18 years old and above, and living in the camp for at least 6 months. We excluded refugees who had serious mental disorders which were identified as any psychological disturbance disabling the participant to complete the interview or give sound responses (1 person), had extreme physical disabilities (6 persons) and those with communication deficiencies like speech and hearing impairments which would make it impossible for them to comprehend the questions being asked (2 persons). We recruited participants between April and May 2017. Six research assistants fluent in English and at least two other languages used by refugees (Swahili, Kirundi, Kinyarwanda, Arabic and Omoro) collected the data. The research assistants were selected among community health workers working within the refugee camp health care system and familiar with the environment. The principal investigator trained the research assistants over 5 days emphasizing the theoretical and practical aspects of the data collection tool, informed consent and participant confidentiality.

### Ethical approval

Ethical approval was obtained from Mbarara University of Science and Technology. Research Ethics committee and the Uganda National Council for Science and Technology. The permission to carry out the study in the camp was obtained from the Office of the Prime Minister. Written informed consent was obtained from the participants and privacy and confidentiality of the participants was ensured. Participants determined to have mental illness were referred to the psychiatric department of Mbarara Regional Referral Hospital for appropriate care.

### Measures

Socio-demographic data were collected using a locally-generated socio-demographic questionnaire and information collected included age, sex, marital status, nationality and education level.

Psychiatric disorders were assessed using the MINI International Neuropsychiatric Interview 7 (MINI). The MINI is a short, structured diagnostic interview compatible with the Diagnostic and Statistical Manual of Mental Disorders 5. It was designed for clinical practice, research in psychiatric primary care settings and epidemiological surveys [19, 20]. The MINI was chosen based on the validity and reliability demonstrated in different populations in Uganda, Brazil, Japan and Europe [21–23].

Perceived needs were assessed using the Humanitarian Emerging Settings Perceived Needs Scale (HESPER) scale developed by the World Health Organization and King’s College London. It explores a wide range of perceived social, psychological and physical needs of people affected by large-scale humanitarian emergencies such as war, conflict or major natural disasters. Pilot-testing of HESPER was done by Semrau et al. [24] in the United Kingdom with Congolese refugees, in Jordan and in Gaza to assess the scale’s feasibility, intelligibility and cultural applicability (2012). The assessment of psychometric properties (i.e. reliability and validity) was done by the same team in Jordan, Haiti and Nepal [24]. HESPER was used in Nepal [25] and in South Sudan [26] to assess psychosocial needs among refugees. HESPER scale contains 26 items on physical, social and psychological needs perceived by respondents as ‘serious problems’. Participants indicated if they perceived each item (Table 3) as a ‘serious problem’ or not. A percentage of respondents who rated the item as a ‘serious problem’ was calculated to understand the magnitude of the need.

The socio-demographic questionnaire, the MINI and the HESPER constituted the data collection tool used in the field.

### Translation

The entire questionnaire was translated into five local languages spoken by all nine nationalities of refugees in the camp (Swahili, Kirundi, Kinyarwanda, Arabic and Omoro) using blind back translation and subsequent corrections by different translators. Translation was done by research assistants under the supervision of the main investigator and a different group of translators back translated into English. The original and the back-translated questionnaires were compared to assess how accurately each item was translated. Items found to be less accurately translated were discussed further to arrive at translations best mirroring the original item.

### Data analysis

The data were analysed using STATA version 13 for both descriptive and inferential analysis. Descriptive statistics were done using univariate analysis to calculate the outcomes (mental disorders) and predictor variables (demographic factors and perceived needs). All qualitative variables were described in the form of frequencies and percentages. We used univariate analysis for sociodemographic factors, perceived needs and psychiatric disorders. We used bivariate analysis to determine the association between socio-demographic risk factors and sex. We used bivariate and multiple logistic regression to determine the association between PTSD and socio-demographic factors, other psychiatric disorders as well as perceived needs using the individual odds ratio (95% confidence intervals). The associations were deemed significant with a *P*-value less than 0.05. Variables were included in multiple regression when they were associated with PTSD at a P-value less than 0.05. Then, we performed a Bonferroni correction [27] to control for the familywise error.

## Results

### Sociodemographic characteristics of participants

We enrolled 387 participants aged 18 years and above. Table 1 shows the demographic characteristics of the participants. Overall, a majority of the participants were females (*n* = 219,56.59%), married (*n* = 188,48.58%), Congolese (*n* = 176, 45.48%) and had attained secondary education (*n* = 207, 53.48%). The mean age was 33.01 (SD 12.2) and the mean duration in the camp was 4.29 years (SD of 3.43).

**Table 1:** Demographic characteristics of the study participants (*n* = 387)

Variables	*N* (%) or Mean (SD)	Male (*n*, %)	Female (*n*, %)
Age	33 (12)	31 (12)	35 (12)
Number of years in Nakivale	4.29 (3.43)	4.09 (3.42)	4.44 (3.44)
Marital Status			
Married	188 (49)	76 (45)	112 (51)
Single	158 (41)	88 (52)	70 (32)
Divorced	8 (2)	0 (0)	8 (4)
Separated	3 (1)	1 (1)	2 (1)
Widowed	30 (8)	3 (2)	27 (12)
Nationality			
Burundian	104 (27)	44 (26)	60 (27)
Congolese	176 (45)	92 (55)	84 (38)
Eritrean	3 (1)	1 (1)	2 (1)
Ethiopian	3 (1)	1 (1)	2 (1)
Rwandese	36 (9)	14 (8)	22 (10)
Somali	64 (16)	15 (9)	49 (22)
Sudanese	1 (0)	1 (1)	0 (0)
Level of Education			
Under Primary	113 (29)	30 (18)	83 (38)
Primary	76 (20)	29 (17)	47 (21)
Secondary	174 (45)	90 (54)	84 (38)
University	24 (6)	19 (11)	5 (2

### Psychiatric disorders among study participants

The most prevalent psychiatric disorder was generalized anxiety disorder (73%), followed by PTSD (67%), major depressive disorder (58%), and substance use disorders (30%). Among participants with PTSD, 70% also met criteria for major depressive disorder, 82% had generalized anxiety disorder and 30% had substance use disorder (Table 2).

**Table 2:** Distribution of Psychiatric disorders among participants

Variables	PTSD (*N*, %)	Major depressive Disorder (*N*, %)	Generalized Anxiety Disorder (*N*, %)	Substance Used disorder (*N*, %)
Sex				
Male	116 (69)	103 (61)	121 (72)	69 (32)
Female	144 (66)	123 (56)	160 (73)	49 (29)
Age				
≤ 20	28 (70)	23 (58)	26 (65)	12 (30)
21–30	106 (63)	87 (51)	124 (73)	36 (21)
31–40	64 (70)	65 (71)	69 (75)	37 (40)
41–50	34 (67)	30 (59)	39 (76)	21 (41)
> 50	28 (80)	21 (60)	23 (66)	12 (34)
Number of years in Nakivale				
≤ 5 years	192 (68)	172 (61)	208 (73)	79 (28)
> 5 years	68 (66)	54 (52)	73 (71)	39 (38)
Marital Status				
Married	131 (70)	112 (60)	139 (74)	62 (33)
Single	100 (63)	90 (57)	111 (70)	48 (30)
Divorced	6 (75)	3 (37)	5 (62)	2 (25)
Separated	3 (100)	3 (100)	3 (100)	2 (67)
Widowed	20 (67)	18 (60)	23 (68)	4 (13)
Nationality				
Burundian	78 (75)	64 (62)	75 (72)	17 (16)
Congolese	115 (65)	91 (52)	131 (74)	45 (26)
Somali	39 (61)	45 (70)	43 (67)	35 (55)
Other nationalities	28 (65)	26 (62)	32 (71)	21 (45)
Level of Education				
under Primary	79 (70)	69 (61)	84 (74)	42 (37)
Primary	49 (64)	46 (61)	56 (74)	21 (28)
Secondary	116 (67)	96 (55)	123 (71)	43 (25)
University	16 (67)	15 (62)	18 (75)	12 (50)
Total	260 (67)	226 (58)	281 (73)	118 (30)

### Perceived needs of the participants

Overall most participants reported having a ‘serious problem’ with almost all the psychosocial perceived needs. The most frequently cited perceived needs were issues related to ‘separation from family members’ and ‘caring for people in the community who are alone’ and ‘distress’. The least reported needs were ‘law and justice in the community’ and ‘moving between places.’ There was strong agreement between men and women in their perception of the most serious psychosocial needs. However women more often reported ‘having a serious problem with safety’ (*P*-value = 0.04) and ‘education for the children’ (P-value = 0.04) (Table 3).

**Table 3:** HESPER Perceived Needs of Participants by gender

Item	*N* (%)	Male (%)	Female (%)	*P*-Value
Do you have a serious problem with …				
Separation from family members	369 (95)	163 (44)	205 (56)	0.12
Care for people who are on their own	362 (94)	159 (44)	203 (56)	0.44
Addressing psychological Distress	355 (92)	157 (44)	198 (56)	0.28
Mental illness in the community	344 (89)	149 (43)	195 (57)	0.91
Safety	334 (86)	152 (46)	182 (54)	0.04
Being displaced from home	333 (86)	140 (42)	193 (58)	0.18
Alcohol or drug use in the community	330 (85)	148 (45)	182 (55)	0.17
Education for the children	330 (85)	136 (41)	194 (59)	0.04
Food	327 (84)	142 (43)	185 (57)	0.99
Income or livelihood	322 (83)	142 (44)	180 (56)	0.54
Respect	318 (82)	140 (44)	178 (56)	0.60
Care for family members	316 (82)	134 (42)	182 (58)	0.40
Healthcare	315 (81)	139 (44)	176 (56)	0.55
The way aid is provided	313 (81)	140 (45)	173 (55)	0.28
Too much free time	307 (80)	131 (43)	176 (57)	0.64
Safety or protection from violence for women	302 (78)	132 (44)	170 (56)	0.65
Physical health	296 (76)	136 (46)	160 (54)	0.07
Place to live in	287 (74)	127 (44)	160 (56)	0.57
Toilets	273 (71)	127 (47)	146 (53)	0.12
Clothes, shoes, bedding or blankets	272 (70)	114 (42)	158 (58)	0.36
Keeping clean	266 (69)	118 (44)	148 (56)	0.58
Drinking water	261 (67)	108 (41)	153 (59)	0.25
Support from others	248 (64)	105 (42)	143 (58)	0.57
Information	196 (51)	90 (46)	106 (54)	0.31
Law and justice in the community	111 (29)	48 (43)	63 (57)	0.97
Moving between places	70 (18)	31 (18)	39 (18)	0.87

### Factors associated with PTSD

The main psychiatric outcomes (major depressive disorder, generalized anxiety disorder, and substance and alcohol use disorders) and socio-demographic factors were run in bivariate analysis with PTSD. A multivariate logistic regression was then conducted including the factors that were significantly associated with PTSD in bivariate analysis. Multivariate logistic regression showed that the odds of having PTSD was 5.13 times higher among participants having substance use disorders [OR = 5.13 (2.32–11.34); *P*-value < 0.0001], 3.27 times higher for those having generalized anxiety disorder [OR = 43.27 (1.85–5.76); P-value < 0.0001] and 4.04 times higher for those having major depressive disorder [OR = 4.04 (2.24–7.30); P-value < 0.0001]. We observed however that being Somali [OR = 0.16 (0.06–0.41); P-value < 0.01] was less associated with PTSD. After application of Bonferroni correction PTSD, substance use disorders, generalized anxiety disorder, major depressive disorder and being Somali remained significantly associated to PTSD. Results are summarized in Table 4.

**Table 4:** Factors associated with PTSD

Factors associated with PTSD	Unadj OR (95% CI)	P-value	Adj OR (95%CI)	P-value
Substance use disorders	6.95 (3.31–14.59)	< 0.0001	5.13 (2.32–11.33)	**< 0.001**
Generalized anxiety disorder	4.05 (2.35–6.98)	< 0.0001	3.27 (1.85–5.76)	**< 0.001**
Major Depressive Disorder	4.11 (2.45–6.91)	< 0.0001	4.04 (2.24–7.30)	**< 0.001**
Marital status				
Married	0.33 (0.05–2.09)	0.24	–	–
Single	0.26 (0.04–1.64)	0.15	–	–
Separated	Reference		Reference	
Widowed	0.25 (0.03–1.86)	0.18	–	–
Nationality				
Burundian	Reference			
Congolese	0.55 (0.28–1.05)	0.07	–	–
Somali	0.16 (0.06–0.40)	< 0.0001	0.16 (0.06–0.41)	**< 0.01**
Other nationalities	0.43 (0.24–1.23)	0.43	–	–
Number of years in the camp				
Below 5 years	Reference			
Above 5 years	1.33 (0.74–2.40)	0.34	–	–
Level of Education				
under Primary degree	Reference		Reference	
Primary degree	0.74 (0.36–1.55)	0.43	–	–
Secondary degree	1.07 (0.55–2.11)	0.84	–	–
University	0.58 (0.20–1.96)	0.38	–	–

Bold indicates correlation is significant at alpha level corrected by sequential Bonferroni method [27]

### Psychosocial perceived needs associated with PTSD

A bivariate and multivariate analysis was used to assess the association between perceived needs and PTSD. In bivariate analysis, many psychosocial perceived needs were associated with PTSD. Most notably, the odds of perceiving ‘distress’ as a ‘serious problem’ is 18 times higher in people with PTSD [OR = 18.10(6.19–52.93); *P*-value < 0.0001]. The other perceived needs associated with PTSD were issues associated with healthcare [OR = 2.46 (1.46–4.15); *P*-value< 0.0001], the way aid is provided [OR = 2.31 (1.38–3.88); *P*-value < 0.0001], or not having a place to live in [OR = 2.29 (1.43–3.67); P-value < 0.0001]. After controlling for confounding factors in a multivariate logistic regression, only the perception of ‘stress’ as a ‘serious problem’ [OR = 6.52 (1.87–9.76); P-value = 0.003], ‘safety and protection for women in the community’ [OR = 2.35 (1.21–4.56); P-value = 0.011], ‘care for family’ [OR = 2.00 (1.10–5.17); P-value = 0.035] and ‘place to live in’ [OR = 1.83 (1.02–3.22); P-value = 0.041] were significantly associated with PTSD at the 0.05 P-value. After the Bonferroni correction, only the perception of ‘distress’ remained significantly associated with PTSD (Table 5).

**Table 5:** HESPER Perceived needs associated with PTSD among refugees in Nakivale camp

Perceived Need	Unadj OR (95% CI)	*P*-Value	Adj OR (95% CI)	*P*-Value
Distress	18.10 (6.19–52.93)	< 0.0001	6.52 (1.87–9.76)	**0.003**
Care for family members	3.66 (2.15–6.22)	< 0.0001	2.00 (1.02–3.22)	0.035
Healthcare	2.46 (1.46–4.15)	< 0.0001	1.21 (0.45–3.57)	0.731
The way aid is provided	2.31 (1.38–3.88)	< 0.0001	1.19 (0.64–3.45)	0.75
Place to live in	2.29 (1.43–3.67)	< 0.0001	1.83 (1.02–3.22)	0.042
Income or livelihood	2.14 (1.25–3.69)	0.01	1.72 (1.02–3.48)	0.048
Safety for women in the community	2.13 (1.30–3.52)	< 0.0001	2.35 (1.21–4.56)	0.011
Education for the children	2.07 (1.17–3.66)	0.01	1.41 (0.64–3.15)	0.39
Physical health	1.88 (1.16–3.06)	0.01	1.12 (0.82–2.21)	0.63
Food	1.86 (1.06–3.25)	0.03	0.86 (0.52–2.23)	0.74
Too much free time	1.85 (1.12–3.08)	0.01	1.15 (0.67–2.22)	0.723
Toilets	1.60 (1.02–2.53)	0.04	1.14 (0.86–2.61)	0.34
Support from others	1.60 (1.03–2.48)	0.03	1.18 (0.91–2.45)	0.54
Safety	1.55 (0.85–2.80)	0.15		
Separation from family members	1.52 (0.60–3.88)	0.38	–	–
Mental illness in the community	1.39 (0.72–2.66)	0.32	–	–
Being displaced from home	1.36 (0.75–2.47)	0.31	–	–
Clothes, shoes, bedding or blankets	1.34 (0.85–2.11)	0.21	–	–
Respect	1.23 (0.76–2.23)	0.34	–	–
Care for people in the community who are on their own	1.16 (0.50–2.71)	0.72	–	–
Alcohol or drug use in the community	1.13 (0.62–2.03)	0.69	–	–
Drinking water	0.98 (0.62–1.54)	0.94	–	–
Information	0.97 (0.63–1.48)	0.88	–	–
Law and justice in the community	0.86 (0.54–1.37)	0.54	–	–
Keeping clean	0.86 (0.54–1.37)	0.52	–	–
Moving between places	0.47 (0.28–0.80)	0.01	0.49 (0.29–1.04)	0.01

Bold indicates correlation is significant at alpha level corrected by sequential Bonferroni method [27]

## Discussion

This study is the first study to assess the prevalence of concurrent major psychiatric illness in Nakivale camp. Furthermore, it is one of few studies systematically assessing perceived needs among refugees in a sub-Saharan African refugee camp. Overall, our results show a high prevalence of PTSD, major depressive disorder, generalized anxiety disorder and substance use among refugees in Nakivale camp. PTSD is highly comorbid with other psychiatric disorders and is associated with perceived psychosocial needs.

A higher prevalence of mental health concerns were identified compared to similar studies among refugees in both pre and post-resettlement settings [28–33]. Two previous studies conducted in Nakivale found relatively high prevalence of PTSD, 61.7% among Congolese refugees and 48.1% among Somali refugees [17, 34]. However, those figures are lower than the prevalence found in this study (67%). It is possible that the differences are due to differences in methodology. First, we used a different tool from previous studies conducted in Nakivale camp. The two previous studies used Post traumatic score diagnostic scale [35] which assesses PTSD symptoms by providing a continuous severity score; the authors set an arbitrary cutoff to distinguish between refugees with and without PTSD. On this point, this scale contrasts with the MINI used in our study which clearly gives results in terms of presence or absence of PTSD according to the core and secondary symptoms described by the DSM V. Secondly, unlike the two previous studies, we included participants from the nine nationalities present in the camp and the tools have been translated in five different languages. It is also possible that the hardship of life has worsened with the changing number of new refugees and limited aid response over time [36]. Finally, it is also possible that these differences in rates of PTSD were due to higher levels of traumatization in different home countries at different times.

In our study, there was a significant association between PTSD and substance use disorder, major depressive disorder and anxiety disorder. Our findings are in agreement with those of previous research which reported a high rate of psychiatric comorbidities in patients with PTSD in refugees living both in Western and African countries. In Norway, it was found that 80% of refugees with PTSD had additional diagnoses such as anxiety disorder, substance use disorders and psychotic disorders [37]. Among Cambodian refugees resettled in the USA, PTSD was highly associated with generalized anxiety disorder [38], and PTSD and major depression were highly comorbid (42% of refugees having both PTSD and MDD) [39]. Among Somali refugees in Nairobi, PTSD was reported to be strongly associated with use of khat [40]. PTSD, depression and anxiety have common overlapping symptoms that can explain to some extent this high association [41, 42]. Flory JD and Yehuda R. have argued that depression is possibly a trauma-related phenotype, a subtype of PTSD [43].

Our results also show a strong association between perceived needs and psychiatric outcomes. The perceived needs associated with PTSD in bivariate analysis were perception of distress, issues associated with care for family members, healthcare, the way aid is provided, place to live in, income or livelihood, safety or protection from violence for women in the community, education for the children, physical health, food, too much free time, toilets and support from others. In multivariate analysis perception of stress as a serious problem, issues with respect to safety and protection for women in the community, care for family and place to live in remained associated with PTSD. After Bonferroni correction, the perception of stress remained significantly associated to PTSD. Existing literature consistently points to the association between daily stressors and the psychiatric outcomes both in refugees resettled in Western countries and those living in African countries [38, 44–48]. In Africa, among Darfur refugees in Chad, lack of access to basic resources and the perception of lack of safety predicted more mental health outcomes than previous exposure to trauma [44]. Among internally displaced persons in Uganda, PTSD was associated with lack of water or food and experiencing illness without medical care [47]. Moreover in a large systematic review concerning low and middle income countries, socioeconomic factors, such as being unemployed, poor living conditions, and being female were associated with poor psychological health in conflict-affected populations [46]. Among Somali refugees of Melkadida camp being deprived of shelter was associated with depression [30]. Among refugees resettled in Western countries, similar observations were made. In Iraqi refugees in Jordan, factors such as inadequate housing, unemployment and change in family structures may play a big role in the occurrence of depression, anxiety disorder and substance use disorders among refugees [25]. Among traumatized Cambodian refugees living in USA, worry about life concerns like lacking of financial resources, children not attending school and health concerns worsened PTSD [38].

In this current study, Somalis presented fewer symptoms of PTSD compared to other populations. The reason for this difference is unclear and should motivate additional studies of proportioned groups of participants. It is possible these differences are due to the fact that the tools were translated in different languages. A difference in livehoods between different nationalities has also been reported in the camp with Somalis being more successful entrepreneurs and having more social cohesion [49].

This body of evidence suggests that unmet perceived needs may play a detrimental role in the psychopathology of PTSD. Furthermore, a study by Bruhn et al. showed clearly how post-migration stressors are complicating treatment of trauma among refugees [50]. Therefore it is important to plan interventions that clearly identify the unmet basic needs associated with more mental outcomes, to address the basic needs, and to integrate them into humanitarian mental health policy.

Unlike some studies which identified being female as associated with poor mental health outcomes [46], our study did not find a significant difference between sex in terms of psychiatric disorders. This lack of difference may reinforce the argument that psychiatric disturbances are connected to the importance of perceived needs in the population. In our study the kind and salience of perceived needs showed a strong similarity between the two sexes, thus, it is possible that the similar prevalence of psychiatric disturbances is connected to these similar perceptions. It is also possible that that there was not a difference in exposure to traumatic events between women and men. A study of Somali and Ethiopian refugees living in the United States identified similarities between sexes with respect to trauma experienced [51].

Thus, high perceived unmet needs are associated with psychiatric outcomes. Complicating this, refugees with mental disturbances are often less able to work and to provide for their needs. Finally, displacement puts refugees in general in a vulnerable position, they are less able to find employment, and therefore cannot independently fulfill their basic needs. The provisions and care provided by aid agencies are not sufficient to fill the large gap of unmet needs.

### Limitations

This study has some limitations. As a cross-sectional study it is not possible to confirm a cause-and-effect relationship between the independent variables and the psychiatric outcomes. In addition, the stratified sampling technique we used may lead to few participants in some minority subgroups. Our limited sample size and the number of variables tested may have impacted the results. Finally, although the tools used to assess psychiatric outcomes and psychosocial perceived needs have been used in Uganda and multiple other African countries, the tools have not been adapted and validated across different nationalities and languages in the camp and this could have contributed to higher prevalences detected.

## Conclusion

This study highlights the high prevalence and co-occurrence of mental disorders among refugees in Nakivale camp and their association with perceived psychosocial needs. The findings suggest that the higher prevalence found in Nakivale camp may be related to the high level of unmet basic needs. Hence, the magnitude and complexity of psychiatric disturbances should mobilize psychosocial support. Furthermore, mental health programs should include a systematic screening and holistic management including clinical approaches and supplementation of basic needs. Interventions aiming at addressing the social needs of refugees are needed in addition to addressing their mental health. Interventions for these populations should therefore include the identification of the basic needs most associated with mental health outcomes and address those that are modifiable. Studies of the long-term impact of such holistic interventions, including longitudinal and, if possible, randomized studies are necessary to continue to build the evidence base for effective approaches to improving the mental health and well-being of refugee populations in Africa and elsewhere.

## Data Availability

The datasets used and/or analysed during the current study are available from the corresponding author on reasonable request.

## Abbreviations

HESPERS: The Humanitarian Emerging Settings Perceived Needs Scale
MINI: Mini International Neuropsychiatric Interview
OR: Odd Ratio
PTSD: Post-Traumatic Stress Disorder
SD: Standard Deviation
UNHCR: United Nation High Commission of Refugee
